# Stuck leaflet after transcatheter aortic valve implantation due to calcium chunk in a patient with bicuspid valve

**DOI:** 10.1093/ehjcr/ytae275

**Published:** 2024-06-07

**Authors:** Hiroto Kawamura, Toru Yoshizaki, Akira Sato

**Affiliations:** Department of Cardiology, Faculty of Medicine, University of Yamanashi, 1110 Shimokato, Chuo, Yamanashi 409-3898, Japan; Department of Cardiology, Faculty of Medicine, University of Yamanashi, 1110 Shimokato, Chuo, Yamanashi 409-3898, Japan; Department of Cardiology, Faculty of Medicine, University of Yamanashi, 1110 Shimokato, Chuo, Yamanashi 409-3898, Japan

## Case description

**Figure 1:**
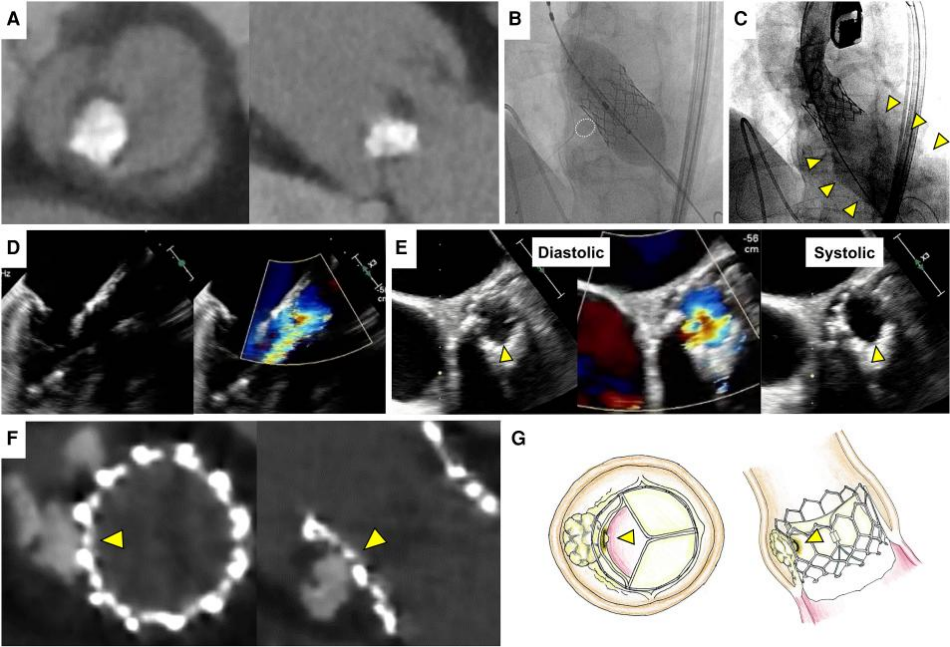
Computed tomography shows a type 0 bicuspid valve with a calcium chunk on the leaflet in the right coronary cusp (*A*). A post-dilation is performed owing to insufficient strut expansion resulting from a calcium chunk (dashed circle) (*B*). Aortography shows severe transvalvular leakage (arrows) after post-dilation (*C*). Transoesophageal echocardiography shows severe transvalvular leakage (*D*); one leaflet on the right coronary cusp side (arrows) is not moving throughout the cardiac cycle, and transvalvular leakage from the same site is observed (*E*). Post-procedural Computed tomography reveals a calcium chunk lodged in the strut of the initial valve (arrows) (*F*). An illustration of a possible mechanism shows the first valve stuck in calcification (arrows) (*G*).

A 64-year-old woman was referred to our hospital with severe aortic stenosis (mean gradient, 57 mmHg). The patient was at high risk of perioperative stroke owing to intracranial artery occlusion. After discussing the treatment plan with a cardiac surgeon and a neurosurgeon, the decision was made to perform extracranial–intracranial bypass surgery after transcatheter aortic valve implantation (TAVI). If valve deterioration occurs, surgical aortic valve replacement will be performed. Computed tomography (CT) revealed a type 0 bicuspid valve with a calcium chunk in the right coronary cusp (RCC) leaflet (*Figure 1A*). Based on an annulus area of 348 mm^2^, an Edwards SAPIEN3 with a 23 mm valve was selected and expanded by 2 mL minus volume. We performed post-dilatation with the same volume because strut expansion was insufficient due to the calcium chunk (*Figure 1B*). The blood pressure had decreased to 60/20 mmHg. Aortography and transoesophageal echocardiography demonstrated severe transvalvular leakage (TVL) (*Figure 1C* and *D*; Supplementary material online, *Videos S1* and *S2*). Transoesophageal echocardiography showed that one leaflet on the RCC side did not move throughout the cardiac cycle, and the TVL from the same site was observed (*Figure 1E*; Supplementary material online, *Videos S3* and *S4*). Emergency valve-in-valve TAVI was performed using an additional 23 mm valve. The TVL completely disappeared, and blood pressure immediately increased to 110/60 mmHg. Post-procedural CT showed a calcium chunk adjacent to the strut of the first valve (*Figure 1F*).

Stuck leaflets are a rare complication with an unclear mechanism.^1^ This case suggests that the calcification of the native valve trapped the leaflet of the first valve after post-dilatation (*Figure 1G*). Post-dilatation is often required in highly calcified cases; however, the possibility of valve deterioration and the need for valve-in-valve TAVI should be considered.^2^

## Supplementary Material

ytae275_Supplementary_Data

## Data Availability

The data underlying this article are available in the article.

## Supplementary material

**Consent:** The authors confirm that written consent for publication of this case report was obtained from the patient in accordance with COPE guidelines.

**Funding:** None declared.
